# Temporal dynamics of shame and guilt in adolescent NSSI: an ambulatory assessment study

**DOI:** 10.3389/fpsyt.2026.1758601

**Published:** 2026-02-17

**Authors:** Andreas Goreis, Rahel L. van Eickels, Dorothy Chang, Diana Klinger, Heidi-Elisabeth Zesch, Karin Prillinger, Bettina Pfeffer, Sofia-Marie Oehlke, Ulrich W. Ebner-Priemer, Laurence Claes, Paul L. Plener, Oswald D. Kothgassner

**Affiliations:** 1 Department of Child and Adolescent Psychiatry, Medical University of Vienna, Vienna, Austria; 2 Comprehensive Center for Pediatrics (CCP), Medical University of Vienna, Vienna, Austria; 3 Comprehensive Center for Clinical Neuroscience and Mental Health (C3NMH), Medical University of Vienna, Vienna, Austria; 4 Department of Clinical and Health Psychology, University of Vienna, Vienna, Austria; 5 Institute of Sports and Sport Sciences, Karlsruhe Institute of Technology, Karlsruhe, Germany; 6 Department of Psychiatry and Psychotherapy, Central Institute of Mental Health, Heidelberg University, Heidelberg, Germany; 7 Clinical Psychology, Faculty of Psychology and Educational Sciences, Katholieke Universiteit (KU) Leuven, Leuven, Belgium; 8 Faculty of Medicine and Health Sciences, University of Antwerp, Antwerp, Belgium; 9 Department of Child and Adolescent Psychiatry and Psychotherapy, University of Ulm, Ulm, Germany

**Keywords:** adolescents, ambulatory assessment, guilt, nonsuicidal self-injury, shame

## Abstract

**Introduction:**

Nonsuicidal self-injury (NSSI) is a common clinical concern among adolescents, yet the roles of shame and guilt as proximal drivers of NSSI in daily life remain unclear. Using an ambulatory assessment design, we examined how these self-conscious emotions relate to momentary NSSI urges and how they fluctuate before and after NSSI acts.

**Methods:**

We recruited 25 adolescents (M = 15.9 years, 72% female, 8% male, 20% gender-diverse) who reported shame, guilt, and NSSI urges four times per day for seven consecutive days on their smartphones. NSSI acts were logged using event-contingent prompts, followed by repeated ratings 10, 20, and 30 minutes after each act. Linear mixed-effects models were used to test concurrent and lagged associations between shame, guilt, and NSSI urges and to estimate event-related changes in these emotions.

**Results:**

Both shame and guilt were positively associated with concurrent NSSI urges at the within- and between-person levels. Shame showed more pronounced temporal variability, with higher levels on Sundays and during the evening hours, and it increased in the minutes following NSSI. Guilt, however, showed no consistent diurnal or weekly pattern and did not change post-NSSI. Neither emotion predicted higher urges at the next prompt one to approximately three hours later.

**Discussion:**

In adolescents who engage in NSSI, shame and guilt co-occur with episodes of elevated urges rather than predict them over short periods. Shame appears more involved in the aftermath of NSSI, consistent with a maintenance process, whereas guilt tracked urges to a similar extent but did not change post-NSSI. Clinically, spikes in both shame and guilt may serve as actionable real-time markers, and post-episode support targeting self-criticism and anticipated judgment may be especially beneficial. Schools and health care settings can reduce barriers to help-seeking by using non-stigmatizing language around NSSI, given that shame frequently drives concealment.

## 1 Introduction

Nonsuicidal self-injury (NSSI), which is typically defined as deliberate destruction of one’s own body tissue without suicidal intent and for purposes that are not socially or culturally sanctioned (1), is a growing clinical challenge in adolescence. It encompasses behaviors such as cutting or burning one’s own body tissue (2), and global prevalence estimates of NSSI among adolescents range from 16% to 17% (3, 4) with lifetime prevalence rates reaching up to 23% (5). Occurrence peaks between ages 15 and 17 and declines in young adulthood (6). NSSI during adolescence is associated not only with mental health problems but also with higher healthcare and economic burdens, reduced productivity, greater morbidity and mortality (7), and an elevated risk of later suicide attempts (8).

Because NSSI is both prevalent and consequential in adolescence, research has established a solid foundation of distal, developmental risk factors, particularly adverse childhood experiences and parental invalidation (9–13). These factors, along with their neurobiological correlates (14), are central to understanding predictors of adolescent NSSI. Yet, when individuals are asked why they engage in NSSI, the most frequently endorsed function is emotion regulation (15), especially the down-regulation of aversive affect (see the seminal work on the Four-Function Model of NSSI, 16, 17). This underscores the need to examine proximal affective states that are directly tied to the immediate intrapersonal function of NSSI. In this context, shame and guilt—distinct yet overlapping self-conscious emotions—remain insufficiently studied as proximal states in NSSI, with notable exceptions (18, 19), and their roles as antecedents and consequences have yet to be clearly specified.

Shame and guilt are self-conscious, discrete emotions elicited by evaluative contexts and oriented toward protecting the social self (20, 21). Shame typically arises from negative self-evaluation or from unwanted exposure of perceived shortcomings (18, 22). In its classic definition, shame reflects a global devaluation of the self rather than an appraisal of a specific behavior (23). Correspondingly, shame is linked to self-scrutiny, withdrawal, a sense of powerlessness, and is often associated with emotional suppression (22). A recent study in adolescents has described an association between shame-proneness and NSSI (13). By contrast, guilt is typically behavior-focused and situational (23). It is therefore more distinct from one’s self-concept or identity and can function as an adaptive emotion-regulation strategy, prompting reparative actions and behavioral change (e.g., apologizing). Although excessive or ruminative guilt can become maladaptive (24, 25), its usual trajectory and functions differ from those of shame, as guilt tends to promote constructive behavior and social cohesion rather than global self-devaluation (e.g., 26).

Beyond these classic distinctions, shame is often conceptualized as comprising both internal shame, reflecting global negative self-evaluation (e.g., feeling flawed or bad as a person), and external shame, reflecting anticipated or perceived negative evaluation by others (e.g., fear of being judged, rejected, or exposed) (27). This distinction may be particularly relevant for NSSI because the behavior is stigmatized and often concealed (28), particularly in adolescent peer contexts, where it can amplify concerns about negative evaluation. Likewise, guilt may range from action-focused, potentially reparative (“adaptive”) guilt to maladaptive forms characterized by excessive self-blame, rumination, or a perceived need for punishment (24), which may overlap with shame in some contexts.

A heightened proneness to shame and guilt has been linked to invalidating caregiving environments and adverse or traumatic childhood experiences (29, 30). These developmental contexts are also commonly associated with NSSI (31) and with a broader spectrum of psychopathology, including depression (32), borderline personality disorder (33, 34), and post-traumatic stress disorder (PTSD, 35; 36). During adolescence, a developmental period characterized by identity formation and increasing salience of peer relationships (37), coping with stressful or negative experiences may be shaped by one’s tendency to experience shame (38) or guilt (39), particularly given that adolescents generally exhibit heightened amygdala responses to social evaluation at the neurobiological level (40). NSSI may serve as a coping mechanism for regulating these self-conscious emotions, whether by managing intense internal states (the intrapersonal function of the Four-Functions model of NSSI; 16), enacting self-punishment, or signaling distress to elicit care or escape aversive social situations (i.e., interpersonal or social-signaling functions, 15). Thus, shame and guilt may constitute theoretically grounded proximal risk factors for the emergence and maintenance of NSSI across its multiple functional pathways. NSSI is also highly stigmatized (41, 42), and guilt and shame may not only precede NSSI but also follow it, adding an additional emotional burden (see, e.g., 43).

A large meta-analysis (19) found that individuals with a history of NSSI reported higher shame (moderate effect, *d* = 0.47) and that shame was positively associated with NSSI frequency (*r* = 0.24, *p* <.05). In the same review, guilt showed no association with NSSI; however, this conclusion was based on only two studies, both conducted with undergraduate samples (44, 45). Subsequent work has largely replicated the positive association between shame and NSSI, but not between guilt and NSSI (e.g., 46), raising the possibility that guilt may be relatively protective against NSSI when used as an emotion-regulation strategy, a pattern also noted for some externalizing outcomes such as criminal offending and substance use (47). At the same time, guilt has been linked to suicidal ideation and attempts, particularly in the context of trauma and PTSD, underscoring its complex and context-dependent role (48). Indeed, among trauma-exposed individuals, forms of guilt such as survivor guilt and moral guilt can heighten self-blame, hopelessness, and a perceived need for punishment (36, 49, 50), patterns that align more closely with suicidal cognition than with the affect-regulatory motives that typically maintain NSSI. This context-dependent nature of guilt underscores the importance of examining it as a situational state in specific contexts, such as NSSI, rather than solely as a stable trait.

Irrespective of the differential associations reported for guilt and shame, a shared limitation is that most studies are cross-sectional, retrospective, and treat these constructs as traits (i.e., guilt/shame proneness, 19). Foundational work has additionally highlighted conceptual and measurement challenges in distinguishing guilt and shame (22). By contrast, ambulatory assessment (repeated sampling on smartphones or other digital devices, see 51) may be more informative because it may capture near-real-time fluctuations in emotions and NSSI thoughts and behaviors in everyday life in an ecologically valid way and enables tests of emotional antecedents and temporal dynamics.

Research on nonsuicidal self-injury (NSSI) and related forms of self-harm has shown that elevations in negative affect are temporally linked to subsequent NSSI urges and, in some studies, to NSSI acts, while negative affect typically declines shortly after NSSI on average (see 52, for a systematic review). In a recent daily life study of 125 young adults with NSSI, the role of self-criticism was examined, and higher levels of self-criticism predicted NSSI urges and behavior within the following two hours (53). Although self-criticism is not synonymous with shame or guilt, these findings underscore that self-defeating cognitions are closely intertwined with NSSI. Another daily life study of 158 adolescents found that shame did not significantly predict subsequent NSSI at the within-person level, but that higher NSSI was associated with increases in shame in between-person analyses (54). Taken together, previous work has already advanced affect-regulation models of NSSI and may inform clinical care, for example via just-in-time digital interventions (45, 55). However, prior studies have either focused on global self-critical cognitions or on shame alone and have not examined shame and guilt side by side as distinct, time-varying states in relation to NSSI urges and acts in adolescents. It therefore remains unclear whether shame and guilt differentially track short-term fluctuations in NSSI urges and show distinct patterns in the immediate aftermath of NSSI episodes, questions that the present study aims to address.

Thus, in the current study we investigated adolescents who had recently engaged in NSSI and assessed momentary shame, guilt, NSSI urges, and NSSI acts in everyday life using ambulatory assessment, sampling four times daily for seven consecutive days and, following NSSI events, at high frequency using event-contingent prompts. We examined within- and between-day patterns of shame and guilt (research question 1). We then tested whether momentary shame and guilt were associated with the concurrent intensity of NSSI urges (research question 2) and whether shame or guilt at the previous prompt predicted NSSI urges at the next assessment (research question 3). Finally, we evaluated how shame and guilt changed when NSSI acts occurred, mapping their trajectories before and after NSSI events (research question 4).

## 2 Method

This study was approved by the Ethics Committee of the Medical University of Vienna (1651/2019) and conducted in accordance with the Declaration of Helsinki. The present manuscript reports additional analyses of data from the cohort described in Goreis et al. (56). Details on study registration and the trial’s sample size and power rationale are provided in Goreis et al. (56) and in the German Clinical Trials Register (DRKS00025905; https://drks.de/search/en/trial/DRKS00025905). The analyses and research questions reported here are novel and have not been published previously.

### 2.1 Participants

We recruited 25 participants (aged 14–18) with NSSI within the last 12 months, primarily patients at the Department of Child and Adolescent Psychiatry, Medical University of Vienna, Austria. The recruitment process utilized the dissemination of posters, social media advertisements, and outreach to psychiatric and psychological practitioners in both the private and public health sectors. To be eligible for the study, participants were required to have a history of at least five NSSI episodes (i.e., self-inflicted damage to body tissue) occurring on at least five separate days within the previous year (i.e., the DSM-5 NSSI disorder frequency criteria). Participants who required acute treatment for conditions such as acute psychosis, acute suicidality, or an immediate risk of harm to themselves or others were excluded. A total of 30 adolescents with NSSI were screened for inclusion. Of these, *n* = 3 were excluded because they did not respond after initial contact or lost interest, and *n* = 2 were excluded because their legal guardian(s) did not provide consent for their child’s participation. There was no dropout after study inclusion.

### 2.2 Procedure

Written informed consent was obtained from participants and their legal guardians before study inclusion. Participants then attended an onboarding visit at the Department of Child and Adolescent Psychiatry, Medical University of Vienna (Austria), where they completed baseline assessments and received training in the ambulatory assessment procedures. Ambulatory monitoring was conducted using the movisensXS application (Movisens GmbH). Participants installed the app on their own smartphones or, when needed, used a study smartphone provided for the study period.

Each participant was also given a study manual that outlined the sampling schedule and provided brief, age-appropriate explanations of the assessed items. Shame was described as feeling bad about oneself, and guilt as feeling bad about a specific action, using everyday language and examples. Given their known overlap in adolescence, these explanations aimed to support consistent interpretation rather than enforce a strict conceptual distinction. Participants were encouraged to ask questions during onboarding, and explanations were clarified as needed before starting the ambulatory assessment. The manual additionally included contact information for questions or technical issues.

The ambulatory assessment began the day after onboarding and continued for seven consecutive days. The 7-day window was chosen to balance sensitivity to day-to-day variation with participant burden. Participants received acoustic prompts to complete brief surveys four times daily: once at a random time between 10:00 am and 11:59 am (morning assessment), and three additional times between 12:00 pm and 8:00 pm (afternoon assessments 1–3) with a minimum interval of 60 minutes between prompts and the option to defer a response by up to 30 minutes. Mean compliance across daily prompts was 65%. In addition to these time-based assessments, participants were instructed to log any NSSI acts via the same app (event sampling). After the monitoring period, participants returned to the lab for a brief debriefing and, when applicable, to return the study phones. All participants received a €25 voucher as compensation, independent of response or compliance rates, and were informed of this during enrolment.

### 2.3 Measures

#### 2.3.1 Baseline

##### 2.3.1.1 Nonsuicidal self-injury

We used the German version of the revised Self-Injurious Thoughts and Behaviors Interview (SITBI-R, 57), a validated, semi-structured interview, to assess NSSI presence, frequency, and characteristics.

##### 2.3.1.2 Perceived stress

The German version (58) of the Perceived Stress Scale (PSS-10) by Cohen et al. (59) was used to indicate perceived stress in the last month. For example, participants were asked, “In the last month, how often have you felt that you were unable to control the important things in your life?”. All items were rated on a 5-point Likert scale (ranging from 0 = never, to 4 = always), with a higher score indicating more perceived stress. The reliability (Cronbach’s α) was .87 in the current sample.

##### 2.3.1.3 Depressive symptoms

We employed the German version (60) of the Beck Depression Inventory II (61) to evaluate depressive symptoms experienced in the preceding two weeks. The BDI-II has well-documented validity and reliability in assessing depressive symptoms in children and adolescents. The BDI-II comprises 21 individual questions, each offering four response options on a Likert scale, ranging from 0 to 3. A higher score on a particular item indicates a more pronounced expression of the symptom. The reliability was α = .93 in the current sample.

##### 2.3.1.4 Post-traumatic stress disorder symptoms

Given the high comorbidity of NSSI with PTSD and the associations between shame/guilt and PTSD symptom severity, we assessed participants’ PTSD symptoms using the German version of the Child and Adolescent Trauma Screen (CATS-2; 62), a self-report instrument for children and adolescents. The CATS-2 comprises 25 items capturing PTSD symptom severity according to ICD-11 and DSM-5 criteria. Items were rated on a 4-point scale (0 = Never, 1 = Sometimes, 2 = Often, 3 = Almost always) referencing the past 4 weeks. Consistent with DSM-5 scoring, scores > 24 were used as a screening threshold for probable PTSD. The internal consistency of the CATS-2 in the present sample was α = .89.

#### 2.3.2 Ambulatory assessment

##### 2.3.2.1 Perceived shame, guilt, and NSSI urges

Momentary shame was assessed with the item “How ashamed do you feel right now?”, and guilt with the item “How guilty do you feel right now?”. The momentary urge to engage in NSSI was measured with the item “How strong is the urge to harm yourself right now?”. The shame and guilt items were adapted from the Positive and Negative Affect Schedule (PANAS; 63). Participants rated each item on a visual analog scale (VAS) ranging from 0 (not at all) to 100 (very much), administered four times per day throughout the ambulatory assessment period. See the **Supplementary Material** for the original German items.

##### 2.3.2.2 NSSI acts

When participants engaged in NSSI, they initiated an event-sampling assessment by activating the movisensXS app. Participants who reported an NSSI act were asked to complete the same perceived shame and guilt items administered during the regular ambulatory assessments repeatedly. They responded to these items immediately after reporting the NSSI act and were then prompted to complete them again 10, 20, and 30 minutes later.

### 2.4 Analyses

Statistical analyses were conducted in R version 4.4.2 using linear mixed-effects models with random intercepts for participants (random slopes were not estimated given the sample size and unbalanced prompts). Momentary observations (level 1) were nested within participants (level 2). Two-sided α was .05; *p*-values used Satterthwaite approximations. Time-varying predictors were decomposed into within-person deviations (person-mean centered) and between-person means; both terms were z-standardized.

For Research Question 1 (dynamics of shame/guilt across and within days), shame and guilt were modeled separately as outcomes with weekday (Monday as reference) and time of day (morning assessment as reference) as fixed effects. For Research Question 2 (shame/guilt as predictors of concurrent NSSI urges), the momentary NSSI urge was predicted from concurrent within-person shame or guilt with between-person means included, adjusting for weekday and time of day. For Research Question 3 (association between shame/guilt and subsequent NSSI urges), we tested whether higher-than-usual shame or guilt at the previous prompt predicted the next urge using lagged within-person models that adjust for each participant’s average shame/guilt and for weekday and time of day, so estimates reflect within-person change over time. Reciprocal models asked whether the previous urge predicted the next shame or guilt and included an autoregressive term for the effect to account for short-term stability and reduce spurious cross-lag associations.

For Research Question 4 (shame/guilt before and after NSSI acts), event-centered models aligned affect to NSSI acts using five levels of time since event (pre, 0, + 10, +20, +30 minutes) and a random intercept for participant; planned contrasts compared each post-event time to pre with Holm adjustment. To examine the robustness of our findings, all models were re-estimated with PTSD symptom severity (CATS-2 total score, grand-mean centered) included as a covariate to account for potential confounding effects of trauma-related symptom severity, given prior evidence linking PTSD symptoms with heightened shame and guilt (36, 64, 65; see also **Supplementary Materials 1**, **2**). We additionally assessed the robustness of concurrent associations (i.e., those of Research Question 2) by controlling for momentary negative affect using a composite negative affect variable (**Supplementary Material 3**). All models used all available data without imputation of missing values.

## 3 Results

The final sample consisted of 25 participants who engaged in NSSI (*M*_age_ = 15.88, 72% female, 8% male, 20% gender-diverse). On average, the sample reported engaging in NSSI 69.4 times in the past year (see **Table 1** for details on NSSI methods/SITBI-R). Eight participants had a prior PTSD diagnosis (according to ICD-10), and dimensional CATS-2 scores for the full sample averaged *M* = 26.68 (*SD* = 16.32), indicating overall high PTSD symptom severity (CATS-2 DSM-5 cut-off: scores >24). Furthermore, as documented in their clinical records, 64% had a preexisting mood disorder, 48% had personality disorders, and 60% were taking psychopharmacological medication (**Table 1**).

**Table 1:** Participant characteristics.

Sample	*M (SD)/N (%)*
Age	15.88 (1.17)
Gender	
Female	18 (72%)
Male	2 (8%)
Gender-diverse	5 (20%)
NSSI 1-week prevalence (SITBI-R)	1.16 (1.82)
NSSI 4-week prevalence (SITBI-R)	5.28 (7.1)
NSSI 1-year prevalence (SITBI-R)	69.4 (62.69)
Perceived Stress (PSS-10, range: 0–40)	27.32 (6.76)
Depressive Symptoms (BDI-II, range: 0–63)	31.96 (13.52)
DSM-5 PTSD Symptoms (CATS-2, range: 0–60)	26.68 (16.32)
Previous diagnoses (ICD-10)	
F3x Mood [affective] disorders	16 (64%)
F4x Neurotic, Stress-Related and Somatoform Disorders	7 (28%)
F5x Behavioral Syndromes Associated with Physiological Disturbances and Physical Factors	1 (4%)
F6x Disorders of Personality and Behavior	12 (48%)
F9x Behavioral and Emotional Disorders	8 (32%)
Current Psychopharmacological Medication	15 (60%)

Range denotes the theoretically possible range of the respective instrument.

### 3.1 NSSI events

In total, 21 NSSI acts were reported by twelve participants during the study period. The most common method was cutting (cuts that do not form specific words/objects/pictures; 13 events), followed by carving (cuts that do form specific words/objects/pictures; 7 events), with one event reported without a specified method. Most NSSI acts (nine) occurred between 4:00 PM and 8:00 PM. Comprehensive information on NSSI characteristics (e.g., triggers, methods, pain ratings) is available in Goreis et al. (56), as these details were not the focus of the current analyses.

### 3.2 Shame

**Figure 1:**
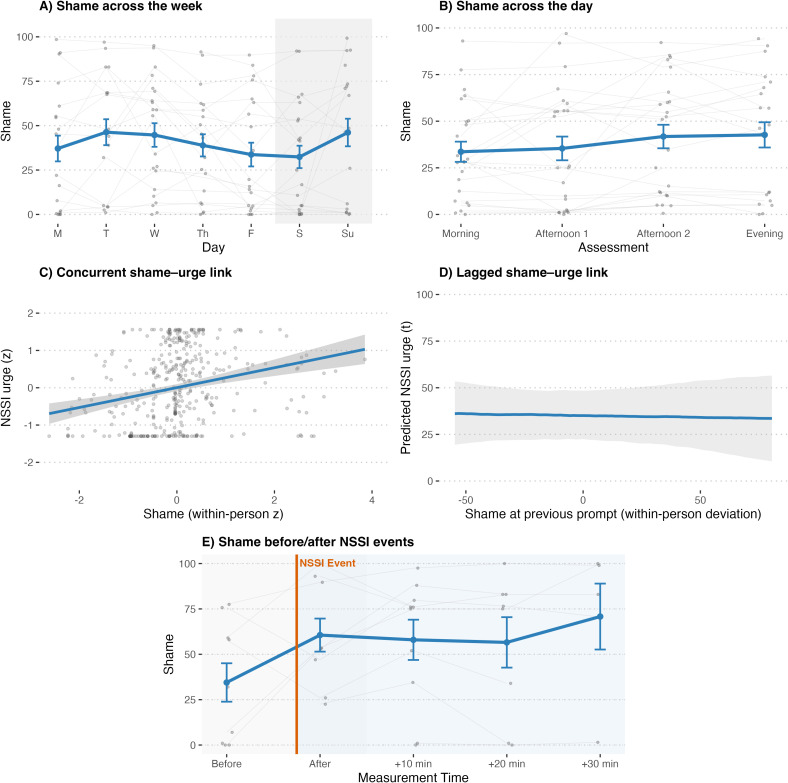
Shame dynamics and associations with NSSI. **(A)** mean shame across weekdays, **(B)** mean shame across the day, **(C)** standardized concurrent within-person association between shame and NSSI urge, **(D)** lagged effect of shame at the previous prompt predicting NSSI urge, and **(E)** shame before and after NSSI events.

Participants reported moderate levels of perceived shame over the week. As shown in **Figure 1A**, relative to Monday, shame was significantly lower on Friday (*b* = −8.64, *SE* = 3.86, *p* = .026) and trended lower on Saturday (*b* = −7.02, *SE* = 3.87, *p* = .070), whereas it was higher on Sunday (*b* = 12.07, *SE* = 3.92, *p* = .002); no differences emerged for Tuesday through Thursday (all *p*s >.190). Across the day (**Figure 1B**), shame did not significantly differ from morning levels at the first afternoon prompt (*b* = 0.45, *SE* = 2.73, *p* = .870), trended higher at the second afternoon prompt (*b* = 5.13, *SE* = 2.82, *p* = .069), and was higher in the evening (*b* = 5.39, *SE* = 2.73, *p* = .049), indicating a modest late-day rise. There was a robust concurrent within-person association between shame and NSSI urge (**Figure 1C**): when participants felt more ashamed than their personal average, they reported stronger urges at that moment (*b* = 0.40, *SE* = 0.05, *t*(332) = 7.36, *p* <.001). At the between-person level, higher mean shame across the week also related to higher overall urges (*b* = 0.71, *SE* = 0.18, *t*(22) = 4.04, *p* <.001). Although time of day and day of week were included as covariates, they did not significantly alter the concurrent association between shame and NSSI urges (all *p*s >.05).

Prospectively (**Figure 1D**), shame at the previous prompt did not predict subsequent urge (*b* = −0.01, *SE* = 0.11, *t*(12) = −0.13, *p* = .900). A reciprocal model indicated that prior urge did not predict later shame (*b* = 0.08, *SE* = 0.10, *t*(11) = 0.89, *p* = .395), while shame exhibited an autoregressive effect (*b* = 0.24, *SE* = 0.07, *t*(179) = 3.29, *p* = .001). In the event-centered model (**Figure 1E**), mean shame level before NSSI was 40.1 (SE = 11.3). Shame did not differ immediately after the act (*p* = .597) but was significantly higher 10 min (*b* = 18.04, *SE* = 8.40, *p* = .036) and 30 min (*b* = 21.83, *SE* = 10.18, *p* = .037) post-event. Levels at 20 min were elevated but not significant (*p* = .107). This pattern indicates that shame increased in the minutes following NSSI rather than decreasing. In sum, shame varies throughout the week (lowest late workweek, highest Sunday) and increases toward the evening. It co-occurs with, but does not prospectively drive, NSSI urges. After NSSI events, shame increases rather than decreases.

### 3.3 Guilt

**Figure 2:**
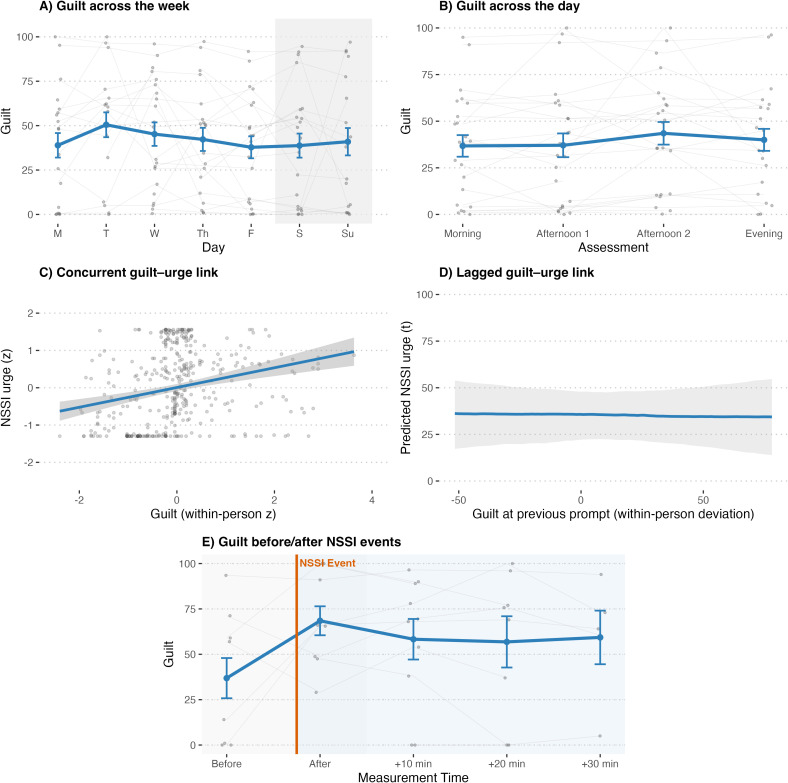
Guilt dynamics and associations with NSSI. **(A)** mean guilt across weekdays, **(B)** mean guilt across the day, **(C)** standardized concurrent within-person association between guilt and NSSI urge, **(D)** lagged effect of guilt at the previous prompt predicting NSSI urge, and **(E)** guilt before and after NSSI events.

Participants also reported moderate levels of perceived guilt across the week. As shown in **Figure 2A**, guilt levels did not differ significantly from Monday on any other day (all *p*s >.050). Although numerically highest on Sunday (*b* = 7.83, *SE* = 4.15, *p* = .060), this difference was not statistically significant, indicating no reliable day-of-week variation in momentary guilt. Guilt also remained stable throughout the day (**Figure 2B**). Compared to the morning assessment, guilt did not differ significantly at the first or second afternoon prompts (*b* = −1.03, *SE* = 2.91, *p* = .720; *b* = 2.91, *SE* = 3.00, *p* = .330) or in the evening (*b* = 0.76, *SE* = 2.91, *p* = .790). Thus, in contrast to shame, momentary guilt showed no systematic diurnal variation. A significant concurrent within-person association emerged between guilt and NSSI urge (**Figure 2C**). When participants felt more guilty than their personal average, they also reported stronger NSSI urges at that moment (*b* = 0.34, *SE* = 0.08, *t*(13) = 4.42, *p* <.001). At the between-person level, participants with higher mean guilt across the week likewise reported higher overall NSSI urges (*b* = 0.77, *SE* = 0.18, *t*(23) = 4.22, *p* <.001). Again, time of day and day of week were not significant predictors in this model (all *p*s >.050).

In the lagged model (**Figure 2D**), guilt at the previous prompt did not predict subsequent NSSI urge (*b* = −0.01, *SE* = 0.11, *t*(14) = −0.07, *p* = .950). In the reciprocal model, prior NSSI urge did not predict subsequent guilt (*b* = −0.04, *SE* = 0.12, *t*(16) = −0.37, *p* = .720), whereas guilt showed a significant autoregressive effect (*b* = 0.37, *SE* = 0.08, *t*(184) = 4.73, *p* <.001), indicating temporal stability of guilt across adjacent time points. In the event-centered model (**Figure 2E**), mean guilt level before NSSI was 44.0 (*SE* = 10.9), and guilt showed no significant changes after NSSI (compared to before NSSI). Immediate post-event levels were slightly higher (*b* = 9.04, *SE* = 8.16, *p* = .273), with similar small, non-significant increases at +10 (*b* = 13.02, *SE* = 8.05, *p* = .112), +20 (*b* = 12.22, *SE* = 8.72, *p* = .167), and +30 minutes (*b* = 10.60, *SE* = 9.76, *p* = .282). In sum, guilt does not vary throughout the week or the day. Similar to shame, it co-occurs with, but does not prospectively drive, NSSI urges. In contrast to shame, guilt remained relatively stable before and after NSSI acts, showing no clear evidence of relief or escalation following self-injury.

### 3.4 Sensitivity analyses adjusting for PTSD symptom severity and negative mood

As a sensitivity check, we re-estimated all models while controlling for PTSD symptom severity (CATS-2 total score, grand-mean centered). Adjusting for PTSD symptom severity increased baseline shame/guilt but did not alter any primary conclusions. In addition, supplementary concurrent models controlling for momentary negative affect yielded comparable results, with within-person associations between shame/guilt and NSSI urges remaining significant. Full estimates are reported in **Supplementary Tables 1–14**.

## 4 Discussion

The present study examined how shame and guilt relate to momentary NSSI urges and acts in adolescents using an ambulatory assessment design. Across seven days, both emotions were positively associated with concurrent NSSI urges at the within-person and between-person levels. Importantly, we found no evidence that guilt was protective and its concurrent association with urges was rather comparable to shame. However, neither shame nor guilt at the previous prompt predicted an increase in the next urge one to three hours later, indicating that these emotions co-occurred with elevated urges rather than temporally preceded them. Finally, and importantly, the high-frequency post-NSSI prompts provided a level of temporal resolution that standard time-based sampling cannot achieve and revealed a clear rise in shame after NSSI acts, whereas guilt showed no significant post-event change. This pattern, which aligns with prior cross-sectional work linking shame more robustly than guilt to self-injurious outcomes (19, 48), suggests that shame may sustain or amplify self-conscious distress following NSSI. All conclusions remained unchanged after adjusting for PTSD symptom severity. Although higher PTSD symptoms were associated with elevated baseline levels of shame and guilt, they did not modify any within-person or event-related effects.

Our first research question examined within-day and between-day dynamics of shame and guilt. Shame fluctuated throughout the week, with lower levels before the weekend and a rise on Sunday as the next school week approached. Within the day, shame was lower in the morning and higher in the evening. These patterns mirror epidemiological evidence that self-injury presentations and suicidal ideation/attempts peak on Sundays and Mondays, with higher levels in the evening and at night (66–69). The reasons for these evening elevations have been hypothesized to reflect a convergence of circadian and contextual risk processes (e.g., greater social isolation and fewer distracting activities later in the day), alongside hormonal changes across the day (e.g., in cortisol or melatonin) that may contribute to heightened evening risk (70, 71). Because most participants were secondary school students, anticipatory school stress may be a plausible explanation for the Sunday increase, we speculate that the expected academic pressure and reduced social structure may have increased negative outcomes.

In line with this, shame and guilt may also accumulate over the course of the day. Ambulatory studies likewise report evening peaks in NSSI urges and suicidal ideation (72–74), consistent with the diurnal trajectory we observed for shame and guilt. Registry evidence points in the same direction: in Ireland, adolescent self-harm/NSSI rates were lowest during school holidays and weekends across more than 150,000 presentations from 2007 to 2019, with similar patterns reported in Canada (69, 75). While school stress is unlikely to be the sole driver of NSSI urges or associated emotions, these temporal regularities suggest a practical window for detection and prevention in school and peer settings.

Regarding our second research question, moments when adolescents experienced more shame or guilt than usual coincided with stronger NSSI urges, and adolescents who generally reported higher levels of these emotions also showed higher average urges. This pattern is consistent with recent ambulatory assessment findings that heightened self-critical thoughts are associated with NSSI urges and behaviors (53), and it seems plausible that self-criticism is intertwined with other self-defeating thoughts and emotions such as shame. Crucially, these links were contemporaneous: elevations in shame or guilt did not forecast higher urges at the next prompt. This pattern, which was highly similar for both shame and guilt, suggests that these emotions function more as proximal markers of urge episodes, and may also be heightened by them, rather than as short-term drivers at the time scale we sampled. Based on our data, we cannot conclude that shame or guilt are feasible predictors of future NSSI urges or acts; however, they appear closely interrelated with urges and tend to co-occur with them. Clinically, even without a prospective effect, spikes in shame or guilt remain actionable as real-time risk markers for support (45, 55), and they should be assessed whenever possible and appropriate.

We further examined shame and guilt in the immediate episodes before and after NSSI acts (research question 4). Here, the two emotions diverged. We found a post-NSSI rise in shame, but not guilt, a pattern consistent with the view that shame is more dynamic in the context of NSSI (19). This pattern fits with a maintenance loop following self-injury rather than a pre-episode trigger, suggesting that shame may sustain the emotional and cognitive conditions that contribute to the recurrence of NSSI. An ambulatory assessment study in adolescents with NSSI likewise reported that regulatory effects, that is, reductions in negative affect following NSSI, did not occur and that NSSI was followed by increases in negative affect immediately and up to one hour afterward (76). Another daily life study in adolescents also reported increases in anger towards the self following higher levels of NSSI, and between-person (but not within-person) increases in NSSI were followed by increased feelings of shame (54). Taken together, these findings suggest that, rather than directly precipitating NSSI, shame intensifies alongside negative affect after the act and may prolong subsequent distress. In other words, in addition to experiencing distress, young people may also feel ashamed of having self-injured, and this response may vary by NSSI function. For example, NSSI motivated by self-punishment may be followed by persistent or heightened shame if the act is perceived as insufficient “atonement,” whereas NSSI serving affect regulation may be associated with lower post-event shame (16, 17). Furthermore, self-injury may also elicit anticipatory shame (e.g., fear of being discovered), thereby amplifying post-event emotional burden.

NSSI still remains one of the most stigmatized mental health concerns (77). Shame, partly driven by this stigma, may become internalized and carry its own psychosocial consequences (41, 42). The stigma surrounding NSSI also has significant negative implications for disclosure and help-seeking, as the behavior is, as mentioned, frequently concealed (see 28, for a review). We encourage clinicians to assess shame, and by extension guilt, given its role as a proximal, concurrent marker of NSSI urges. We also reiterate prior calls to use respectful, non-stigmatizing language when discussing NSSI (e.g., 78; see also 79, for concrete examples applicable to clinical care). This stance can reduce stigma and, in turn, mitigate the experience of shame among those who self-injure.

Importantly, such efforts should not be confined to clinical or school settings. Shame related to NSSI may also be shaped and reinforced within families, peer groups, and digital environments, including through critical parenting responses, peer reactions, or exposure to stigmatizing narratives online (13, 25, 43). Addressing these broader contexts may therefore be critical for reducing the emergence and maintenance of shame. At a public health level, schools can train staff in non-stigmatizing language and responses and provide clear pathways to confidential help. Stigma reduction campaigns that use person-first language and normalize help-seeking (e.g., 41) may reduce shame and, in turn, lower barriers to disclosure and care.

### 4.1 Future research

Future research should, as mentioned, test whether targeting shame-relevant contexts and triggers across adolescents’ everyday environments, alongside real-time, low-threshold support (e.g., ecological momentary or digitally delivered interventions), can reduce shame and interrupt escalation around urge episodes and after NSSI acts (see 55, for a review). Together, these steps may help translate the temporal and affective signatures identified in this study into concrete opportunities for prevention and intervention. Future research could also incorporate qualitative or mixed-method designs to examine how adolescents understand and differentiate shame and guilt in relation to NSSI, and whether these emotions take distinct forms before versus after urges or acts. Furthermore, we highlight the importance of incorporating brief functional assessments to capture the primary motive for NSSI at each event-related prompt. Such work may help refine ambulatory assessment items to better capture the breadth and nuance of these experiences.

### 4.2 Limitations

This study has several limitations. Mean levels of shame and guilt were in the moderate range, consistent with the psychological strain expected in this sample, and most participants were receiving mental health care. This profile, however, does not guarantee representativeness of adolescents or young adults who self-injure outside clinical settings. Comorbidities common in clinical samples, including borderline personality symptoms linked to elevated shame (34), may also have influenced the results observed here. Shame and guilt were assessed with single-item self-reports at prompts spaced several hours apart, meaning that very rapid fluctuations, including those occurring overnight, may have been missed. While this approach reduced burden and supported ecological validity, these single-item ratings likely captured global momentary feelings and could not distinguish internal versus external shame or adaptive versus maladaptive guilt (future ambulatory studies could incorporate brief multi-item state measures). Furthermore, our operationalization of shame (as self-focused) and guilt (as behavior-focused) may not fully align with how adolescents use these emotion labels in everyday life. Trait measures partly circumvent this issue by not explicitly naming the emotion, whereas our state items did. The 1 to 3 hour interval between prompts may have been too coarse to detect shorter-term temporal effects. Additionally, the sample size was modest, albeit comparable to other daily-life studies in adolescents who engage in NSSI (see 80), which limits precision and reduces generalizability. Compliance was moderate (65%), and missingness may have introduced bias if nonresponse was systematically related to our outcomes and/or predictors. Lastly, NSSI acts were relatively infrequent, which reduced statistical power for event-aligned analyses beyond the limitations imposed by the overall modest sample size and rendered post-act estimates sensitive to a small number of individuals. However, this limitation is largely inherent to event-based ambulatory assessment designs targeting low-frequency behaviors such as NSSI.

## 5 Conclusion

In conclusion, our study provides novel, ecologically valid insights into how shame and guilt unfold around NSSI in adolescents’ daily lives. Both emotions were concurrently associated with stronger urges, yet neither predicted higher urges at the next prompt (i.e., within a 1 to 3 hour interval). Overall, our findings suggest that shame is more involved in the aftermath and potential maintenance of distress than in short-term triggering of new NSSI urges, and guilt tracked urges concurrently but showed no evidence of a protective or prospective association in this design. Clinicians should routinely assess shame and guilt, treat elevations in these emotions as potential real-time risk markers, and strengthen skills plans not only for managing urges but also for the hours following an episode. This may include strategies targeting self-criticism and anticipated judgment (see 81, 82, for treatment options), with Dialectical Behavior Therapy, which emphasizes emotion regulation and self-validation, being particularly well suited in this context.

## Data Availability

The raw data supporting the conclusions of this article will be made available by the authors, without undue reservation.
