# Supplementary material for: Temporal dynamics of shame and guilt in adolescent NSSI: an ambulatory assessment study
Source: Front Psychiatry. 2026 Feb 17;17:1758601. doi: 10.3389/fpsyt.2026.1758601 (PMC12953566; doi:10.3389/fpsyt.2026.1758601)
Supplement: Supplementary file 1 [file Table1.docx]

Supplementary Material

# Supplementary Analyses: Shame (Including PTSD Symptom Severity)

**Supplementary Table 1. Multilevel model of within-day shame fluctuations (time of day).**

| **Predictor** | ***b*** | ***SE*** | ***t*** | ***p*** |
| --- | --- | --- | --- | --- |
| Intercept | 35.71 | 5.73 | 6.23 | <.001 |
| Afternoon 1 | 0.52 | 2.73 | 0.19 | .850 |
| Afternoon 2 | 5.22 | 2.82 | 1.85 | .065 |
| Afternoon 3 | 5.48 | 2.73 | 2.01 | .046 |
| CATS-2 (grand-mean centered) | 0.75 | 0.34 | 2.21 | .037 |

Note. Reference = morning assessment. Within-person variation modeled across the day.

**Supplementary Table 2. Multilevel model of between-day shame fluctuations (weekday).**

| **Predictor** | ***b*** | ***SE*** | ***t*** | ***p*** |
| --- | --- | --- | --- | --- |
| Intercept | 41.17 | 6.11 | 6.73 | <.001 |
| Tuesday | 0.38 | 4.19 | 0.09 | .927 |
| Wednesday | 4.95 | 3.89 | 1.27 | .205 |
| Thursday | -3.83 | 3.77 | -1.02 | .310 |
| Friday | -8.57 | 3.86 | -2.22 | .027 |
| Saturday | -6.86 | 3.87 | -1.77 | .077 |
| Sunday | 12.15 | 3.92 | 3.10 | .002 |
| CATS-2 (grand-mean centered) | 0.74 | 0.34 | 2.16 | .042 |

Note. Reference = Monday. Within-person variation modeled across weekdays.

**Supplementary Table 3. Concurrent association between shame and NSSI urge.**

| **Predictor** | ***b*** | ***SE*** | ***t*** | ***p*** |
| --- | --- | --- | --- | --- |
| Intercept | 12.02 | 9.81 | 1.23 | .232 |
| Shame (within-person) | 0.40 | 0.06 | 7.35 | <.001 |
| Shame (between-person) | 0.79 | 0.20 | 4.01 | .001 |
| CATS-2 (grand-mean centered) | -0.31 | 0.34 | -0.92 | .368 |

Note. Within-person shame = person-mean centered; between-person shame = participant mean.

**Supplementary Table 4. Lagged association: prior shame predicting subsequent NSSI urge.**

| **Predictor** | ***b*** | ***SE*** | ***t*** | ***p*** |
| --- | --- | --- | --- | --- |
| Intercept | -0.25 | 11.18 | -0.02 | .982 |
| Shame (within-person) | 0.86 | 0.22 | 3.94 | .001 |
| Lagged Shame (between-person) | -0.03 | 0.11 | -0.31 | .763 |
| Lagged Urge (within-person) | 0.38 | 0.08 | 4.95 | <.001 |
| CATS-2 (grand-mean centered) | -0.39 | 0.39 | -1.00 | .328 |

Note. Lagged shame (t–1) predicts next NSSI urge (t); within-person predictors person-mean centered.

**Supplementary Table 5. Reciprocal lagged association: prior NSSI urge predicting subsequent shame.**

| **Predictor** | ***b*** | ***SE*** | ***t*** | ***p*** |
| --- | --- | --- | --- | --- |
| Intercept | 11.35 | 9.28 | 1.22 | .232 |
| Urge (within-person) | 0.64 | 0.17 | 3.76 | .001 |
| Lagged Urge (within-person) | 0.13 | 0.08 | 1.57 | .179 |
| Lagged Shame (between-person) | 0.23 | 0.07 | 3.28 | .001 |
| CATS-2 (grand-mean centered) | 0.92 | 0.29 | 3.19 | .009 |

Note. Lagged NSSI urge (t–1) predicts next shame (t); within-person predictors person-mean centered.

**Supplementary Table 6. Event-centered model of shame before and after NSSI acts.**

| **Predictor** | ***b*** | ***SE*** | ***t*** | ***p*** |
| --- | --- | --- | --- | --- |
| Intercept | 42.13 | 11.10 | 3.80 | .002 |
| Immediately After Event | 3.50 | 8.54 | 0.41 | .684 |
| +10 min after | 17.16 | 8.41 | 2.04 | .046 |
| +20 min after | 13.94 | 9.11 | 1.53 | .132 |
| +30 min after | 21.07 | 10.18 | 2.07 | .044 |
| CATS-2 (grand-mean centered) | 0.75 | 0.57 | 1.32 | .217 |

Note. Reference = pre-event. Within-person variation modeled across NSSI event timeline.

# Supplementary Analyses: Guilt (Including PTSD Symptom Severity)

**Supplementary Table 7. Multilevel model of within-day guilt fluctuations (time of day).**

| **Predictor** | ***b*** | ***SE*** | ***t*** | ***p*** |
| --- | --- | --- | --- | --- |
| Intercept | 38.82 | 5.48 | 7.08 | <.001 |
| Afternoon 1 | -0.94 | 2.91 | -0.32 | .748 |
| Afternoon 2 | 3.04 | 3.00 | 1.01 | .312 |
| Afternoon 3 | 0.90 | 2.91 | 0.31 | .758 |
| CATS-2 (grand-mean centered) | 0.79 | 0.32 | 2.47 | .022 |

Note. Reference = morning assessment. Within-person variation modeled across the day.

**Supplementary Table 8. Multilevel model of between-day guilt fluctuations (weekday).**

| **Predictor** | ***b*** | ***SE*** | ***t*** | ***p*** |
| --- | --- | --- | --- | --- |
| Intercept | 41.64 | 5.76 | 7.23 | <.001 |
| Tuesday | 0.83 | 4.44 | 0.19 | .852 |
| Wednesday | 5.72 | 4.12 | 1.39 | .166 |
| Thursday | -2.03 | 3.99 | -0.51 | .611 |
| Friday | -5.52 | 4.09 | -1.35 | .178 |
| Saturday | -4.00 | 4.10 | -0.98 | .329 |
| Sunday | 7.93 | 4.15 | 1.91 | .057 |
| CATS-2 (grand-mean centered) | 0.79 | 0.31 | 2.53 | .019 |

Note. Reference = Monday. Within-person variation modeled across weekdays.

**Supplementary Table 9. Concurrent association between guilt and NSSI urge.**

| **Predictor** | ***b*** | ***SE*** | ***t*** | ***p*** |
| --- | --- | --- | --- | --- |
| Intercept | 6.18 | 10.12 | 0.61 | 0.547 |
| Guilt (within-person) | 0.34 | 0.05 | 6.81 | <.001 |
| Guilt (between-person) | 0.9 | 0.2 | 4.42 | <.001 |
| CATS-2 (grand-mean centered) | -0.44 | 0.34 | -1.3 | .205 |

Note. Within-person guilt = person-mean centered; between-person guilt = participant mean.

**Supplementary Table 10. Lagged association: prior guilt predicting subsequent NSSI urge.**

| **Predictor** | ***b*** | ***SE*** | ***t*** | ***p*** |
| --- | --- | --- | --- | --- |
| Intercept | -12.56 | 11.28 | -1.11 | .276 |
| Lagged Guilt (within-person) | -0.06 | 0.11 | -0.52 | .612 |
| Guilt (between-person) | 1.11 | 0.22 | 5.09 | <.001 |
| Lagged Urge (within-person) | 0.33 | 0.07 | 4.49 | <.001 |
| CATS-2 (grand-mean centered) | -0.80 | 0.35 | -2.27 | .042 |

Note. Lagged guilt (t–1) predicts next NSSI urge (t); within-person predictors person-mean centered.

**Supplementary Table 11. Reciprocal lagged association: prior NSSI urge predicting subsequent guilt.**

| **Predictor** | ***b*** | ***SE*** | ***t*** | ***p*** |
| --- | --- | --- | --- | --- |
| Intercept | 14.48 | 8.35 | 1.74 | .093 |
| Lagged Urge (within-person) | -0.02 | 0.11 | -0.15 | .885 |
| Urge (between-person) | 0.57 | 0.15 | 3.84 | .001 |
| Lagged Guilt (between-person) | 0.35 | 0.08 | 4.57 | .001 |
| CATS-2 (grand-mean centered) | 0.71 | 0.27 | 2.60 | .018 |

Note. Lagged NSSI urge (t–1) predicts next guilt (t); within-person predictors person-mean centered.

**Supplementary Table 12. Event-centered model of guilt before and after NSSI acts.**

| **Predictor** | ***b*** | ***SE*** | ***t*** | ***p*** |
| --- | --- | --- | --- | --- |
| Intercept | 46.39 | 10.4 | 4.46 | <.001 |
| Immediately After Event | 7.89 | 8.18 | 0.96 | .339 |
| +10 min after | 11.96 | 8.06 | 1.48 | .144 |
| +20 min after | 11.06 | 8.72 | 1.27 | .210 |
| +30 min after | 9.69 | 9.75 | 0.99 | .325 |
| CATS-2 (grand-mean centered) | 0.88 | 0.53 | 1.65 | .128 |

Note. Reference = pre-event. Within-person variation modeled across NSSI event timeline.

# Supplementary Analysis: Negative Affect as a Covariate in Concurrent Shame/Guilt–Urge Associations

To examine whether the concurrent associations between shame/guilt and NSSI urges were primarily driven by general negative affect, we re-estimated the models including a composite negative affect variable assessed concurrently with shame and guilt. This variable was derived from four items (anxious, nervous, irritable, and hostile) of the Positive and Negative Affect Schedule–Short Form (PANAS-SF), capturing those momentary feelings which were assessed at the same prompts as our other variables.

In those two concurrent models adjusting for negative affect at the within- and between-person level, both momentary shame (β = 0.222, *p* < .001) and momentary guilt (β = 0.176, *p* < .001) remained positively associated with NSSI urges, whereas the between-person components of shame and guilt were not significant. Thus, these findings suggest that the concurrent links between shame/guilt and NSSI urges cannot be explained solely by general negative affect, but reflect additional emotion-specific variance at the momentary level.

**Supplementary Table 13. Concurrent association between shame and NSSI urge.**

| **Predictor** | ***b*** | ***SE*** | ***t*** | ***p*** |
| --- | --- | --- | --- | --- |
| Intercept | 0.09 | 7.65 | 0.01 | .991 |
| Shame (within-person) | 0.22 | 0.06 | 4.02 | < .001 |
| Shame (between-person) | −0.31 | 0.28 | −1.11 | .280 |
| Negative affect (within-person) | 0.57 | 0.07 | 7.80 | < .001 |
| Negative affect (between-person) | 1.31 | 0.32 | 4.09 | < .001 |

Note. Within-person shame/negative affect = person-mean centered; between-person shame/negative affect = participant mean.

**Supplementary Table 14. Concurrent association between guilt and NSSI urge.**

| **Predictor** | ***b*** | ***SE*** | ***t*** | ***p*** |
| --- | --- | --- | --- | --- |
| Intercept | 0.58 | 7.85 | 0.07 | .942 |
| Guilt (within-person) | 0.18 | 0.05 | 3.47 | < .001 |
| Guilt (between-person) | −0.07 | 0.27 | −0.24 | .813 |
| Negative affect (within-person) | 0.59 | 0.07 | 7.98 | < .001 |
| Negative affect (between-person) | 1.05 | 0.30 | 3.56 | .002 |

Note. Within-person shame/negative affect = person-mean centered; between-person shame/negative affect = participant mean.

# Supplementary Analysis: Depressive Symptoms and Compliance

Compliance with daily prompts may have been associated with individual depressive symptom burden at baseline. In a supplementary analysis restricted to participants with missing ambulatory assessment data (*n* = 22), the absolute number of missed prompts per participant was strongly positively correlated with depressive symptom severity as assessed by the Beck Depression Inventory–II (*r* = .60, *p* = .003). Accordingly, it cannot be ruled out that prompts were disproportionately missed during periods of heightened distress, representing an important limitation when interpreting the temporal dynamics of affect and urges in this sample.

# Translation of Items (German Version)

Perceived shame: "Wie beschämt fühlst du dich gerade?"

Perceived guilt: "Wie schuldig fühlst du dich gerade?"

NSSI urge: "Wie stark ist gerade der Drang, dich selbst zu verletzen?“
